# Structured reporting of brain MRI following mechanical thrombectomy in acute ischemic stroke patients

**DOI:** 10.1186/s12880-021-00621-4

**Published:** 2021-05-25

**Authors:** Sebastian Mönch, Tiberiu Andrisan, Kathleen Bernkopf, Benno Ikenberg, Benjamin Friedrich, Claus Zimmer, Dennis M. Hedderich

**Affiliations:** 1 Department of Diagnostic and Interventional Neuroradiology, Klinikum Rechts Der Isar, School of Medicine, Technical University Munich, Ismaninger Straße 22, 81675 Munich, Germany; 2 Department of Radiology, University Hospital, LMU Munich, Munich, Germany; 3 Department of Neurology, Klinikum Rechts Der Isar, School of Medicine, Technical University Munich, Munich, Germany

**Keywords:** Structured reporting, Quality assessment, Magnetic resonance imaging, Acute ischemic stroke, Mechanical thrombectomy

## Abstract

**Background:**

To compare the quality of free-text reports (FTR) and structured reports (SR) of brain magnetic resonance imaging (MRI) examinations in patients following mechanical thrombectomy for acute stroke treatment.

**Methods:**

A template for SR of brain MRI examinations based on decision trees was designed and developed in house and applied to twenty patients with acute ischemic stroke in addition to FTR. Two experienced stroke neurologists independently evaluated the quality of FTR and SR regarding clarity, content, presence of key features, information extraction, and overall report quality. The statistical analysis for the differences between FTR and SR was performed using the Mann–Whitney U-test or the Chi-squared test.

**Results:**

Clarity (p < 0.001), comprehensibility (p < 0.001), inclusion of relevant findings (p = 0.016), structure (p = 0.005), and satisfaction with the content of the report for immediate patient management (p < 0.001) were evaluated significantly superior for the SR by both neurologist raters. One rater additionally found the explanation of the patient’s clinical symptoms (p = 0.003), completeness (p < 0.009) and length (p < 0.001) of SR to be significantly superior compared to FTR and stated that there remained no open questions, requiring further consultation of the radiologist (p < 0.001). Both neurologists preferred SR over FTR.

**Conclusions:**

The use of SR for brain magnetic resonance imaging may increase the report quality and satisfaction of the referring physicians in acute ischemic stroke patients following mechanical thrombectomy.

*Trial registration* Retrospectively registered.

## Background

Ischemic stroke is a leading cause of disability and mortality worldwide [1]. Recently, mechanical thrombectomy (MT) has been proven to be highly beneficial for clinical outcomes of acute ischemic stroke patients (AIS) with an underlying large vessel occlusion [2–6]. In addition to this revolution in AIS therapy, great efforts have been put into individualizing rehabilitation measures following stroke unit or intensive care treatment [7]. In addition to the clinical assessment, specific knowledge about the affected brain regions and possible complications, such as hemorrhage or brain edema, is essential for clinical decision-making and thus for the patient’s prognosis and grade of disability. Magnetic resonance imaging (MRI) of the brain is a mainstay in neuroradiological practice and is essential for the assessment of stroke. To date, the vast majority of these radiology reports are produced in free-text report (FTR) format, describing narrative reports which were typed manually or dictated into voice recording systems. FTR are known to be associated with excessive variability in language, clarity, and content [8]. Thus, report quality may be reduced, making it potentially more difficult for referring clinicians to identify key points necessary for patient care. Structured reports (SR), are typically based on systematic checklist and decision tree options using specifically designed templates and have gained much attention recently. By minimizing shortcomings of FTR disadvantages and serving as an ideal basis for artificial intelligence applications, they have been introduced into clinical practice lately [8, 9]. For example, SR of brain MRI examinations where shown to increase the rate of included disease-relevant findings in Multiple Sclerosis patients and were preferred by clinicians [10]. We know from previous studies that SR increases report completeness and attention to detail [11, 12]. As stated above, detailed and clear descriptions of affected brain regions are of high clinical interest in subacute stroke patients. Thus, we hypothesized that SR would be favorable over FTR in patients after MT. We addressed this question in a single-center, retrospective study.

## Methods

### Study design

In this monocentric, retrospective study, consecutive AIS patients from daily clinical routine who had been treated with MT and had an MRI report of the brain from two neuroradiologists (S.M. or D.M.H.) three to five days after this procedure were included from 08/2017 until 03/2018. Informed consent was waived due to the retrospective nature of the study by the ethics committee of the Technical University of Munich. All procedures were approved by the ethics committee of the Technical University of Munich (application number 56/20 S-KH). All methods were carried out in accordance with the Declaration of Helsinki.

### Endovascular Intervention

Patients were eligible for MT if CT angiography (CTA) confirmed a large vessel occlusion of the internal carotid artery, the middle cerebral artery or the basilar artery. Parenchymal infarction was limited to an Alberta Stroke Program Early CT Score (ASPECTS) of > 5 in middle cerebral artery strokes. No age or perfusion selection was applied within the timeframe of 6 h. Patients beyond the time window of 6 h since symptom onset or with unknown/wake-up symptom onset were selected by a target mismatch on CT Perfusion according to local standard operating procedures. For basilar artery occlusions no time window was applied. After a femoral access was established a stent-retriever based MT was performed. Hemorrhagic transformation of ischemic infarct were defined according to ECASS II-classification [13]. Successful recanalization was defined as an extended Thrombolysis in cerebral infarction score of greater than or equal to 2b. Procedure related complications were defined as arterial dissection, intracranial vessel perforation or subarachnoid hemorrhage on control CT imaging. Intravenous thrombolysis was performed by weight adapted tissue plasminogen activator according to current guidelines.

### Brain MRI

The brain MRI scanning protocol included axial fluid attenuation inversion recovery (FLAIR), axial T2*, axial T2, and 3D arterial time-of-flight MR angiography (art. TOF) and axial diffusion weighted imaging (DWI) sequences (Table 1). All scans were performed using a 3.0 T MRI system (Philips Achieva dStream 3.0 T, Philips Healthcare, Best, Netherlands) equipped with a 32-channel phased array head coil (Philips) for signal reception.

**Table 1:** Sequence parameter specifications

Sequence	TE (ms)	TR (ms)	TI (ms)	AT (s)
Ax. FLAIR	140.0	12,000	2850.0	180
Ax. T2	80.0	3200	/	166
Ax. T2*	16.1	858 ("shortest")	/	147
3D art. TOF	3.5	25	/	360
Ax. DWI	55.0	10,126	/	366

*TE* time to echo, *TR* time to repeat, *TI* inversion time, *AT* acquisition time, / not applicable, *Ax.* axial, *FLAIR* fluid attenuated inversion recovery, *art. TOF* arterial time-of-flight MR angiography, *DWI* diffusion weighted imaging

### Structured reporting template for stroke MRI

**Fig. 1:**
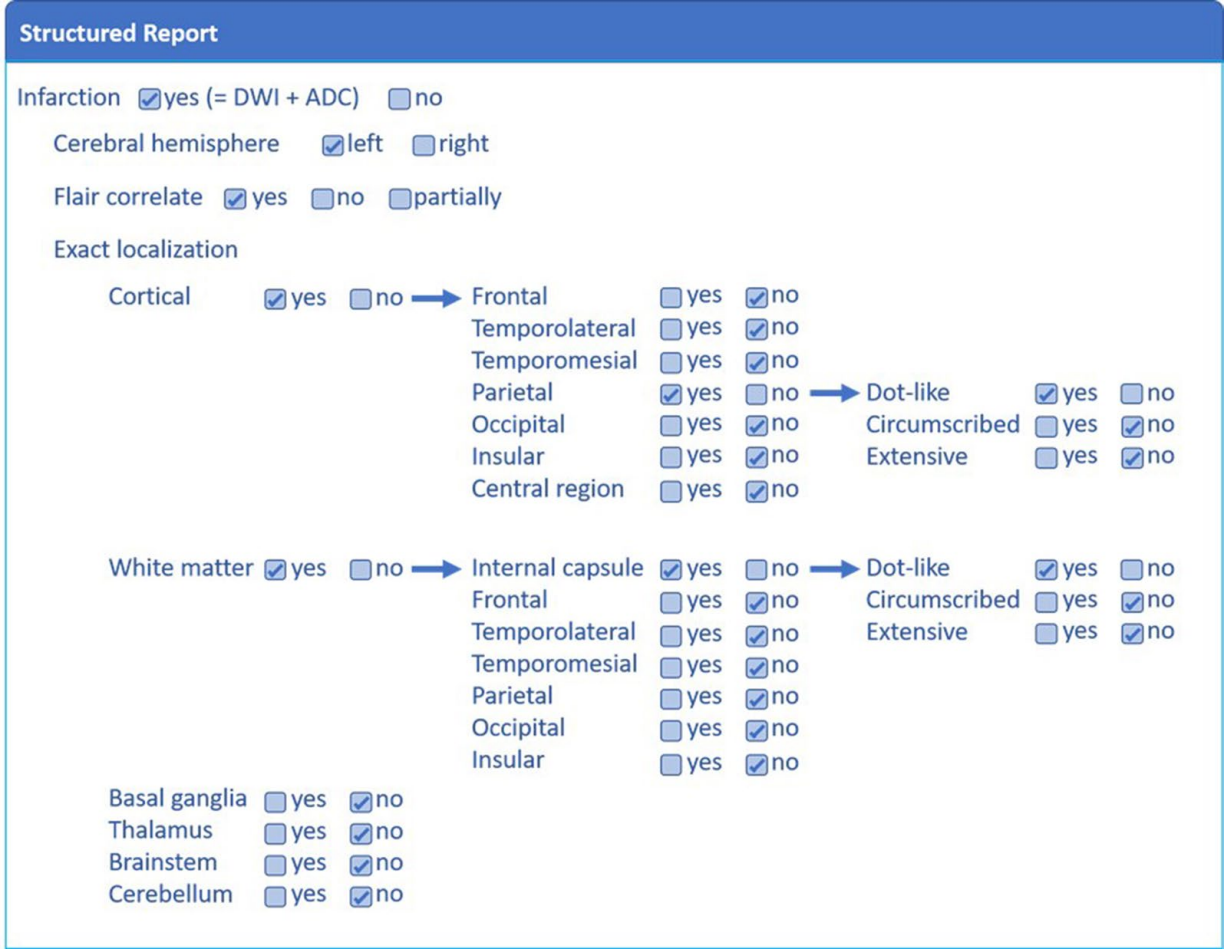
Conceptual example of the clickable decision tree of the structured reporting template

The content and design of the SR template was performed by neuroradiologists (S.M., D.M.H.). The SR template was developed in house. In principle, this software allows the radiologist to select check boxes containing subitems based on decision trees (excerpt of this template shown in Fig. 1). These clickable decisions are then automatically transferred into predefined text phrases. The SR template additionally offered the option to manually add sentences in dedicated free-text fields.

Next to the appearance on MRI sequences the details on infarct location and extent are subitems to be selected by the radiologist in order for the software to concomitantly generate the report. Abbreviations: DWI = Diffusion weighted imaging, ADC = Apparent diffusion coefficient, FLAIR = Fluid-attenuated inversion recovery.

The SR consisted of a findings section and an impressions section. In the findings section, the radiologist had to provide information regarding initially occluded vessel, presence of stenting procedures, intravenous lysis administration, recanalization success, MRI sequences, infarction (diffusion restriction, mismatch between diffusions weighted image and FLAIR image, extent (dot-like, circumscribed, extensive), localization (frontal, temporolateral, temporomesial, parietal, occipital, insular, central region), affection (cortex, white matter, basal ganglia, thalamus, cerebellum or brain stem), complications (e.g. intracranial hemorrhage, hemorrhagic transformation of ischemic infarct, infarct swelling, obstructive hydrocephalus, midline shift, herniation), status of recanalized vessel (stenosis, persisting occlusion) and presence of other occluded vessels, microangiopathy, and older infarcts. This information was then automatically transferred and summarized in the impression section.

### Free-text reports

As FTR, the original, already existing report generated manually or by using a voice dictating system were used. All FTRs were produced by the same radiologist as the SR and proofread by a neuroradiology consultant in clinical routine.

### Report evaluation

**Fig. 2:**
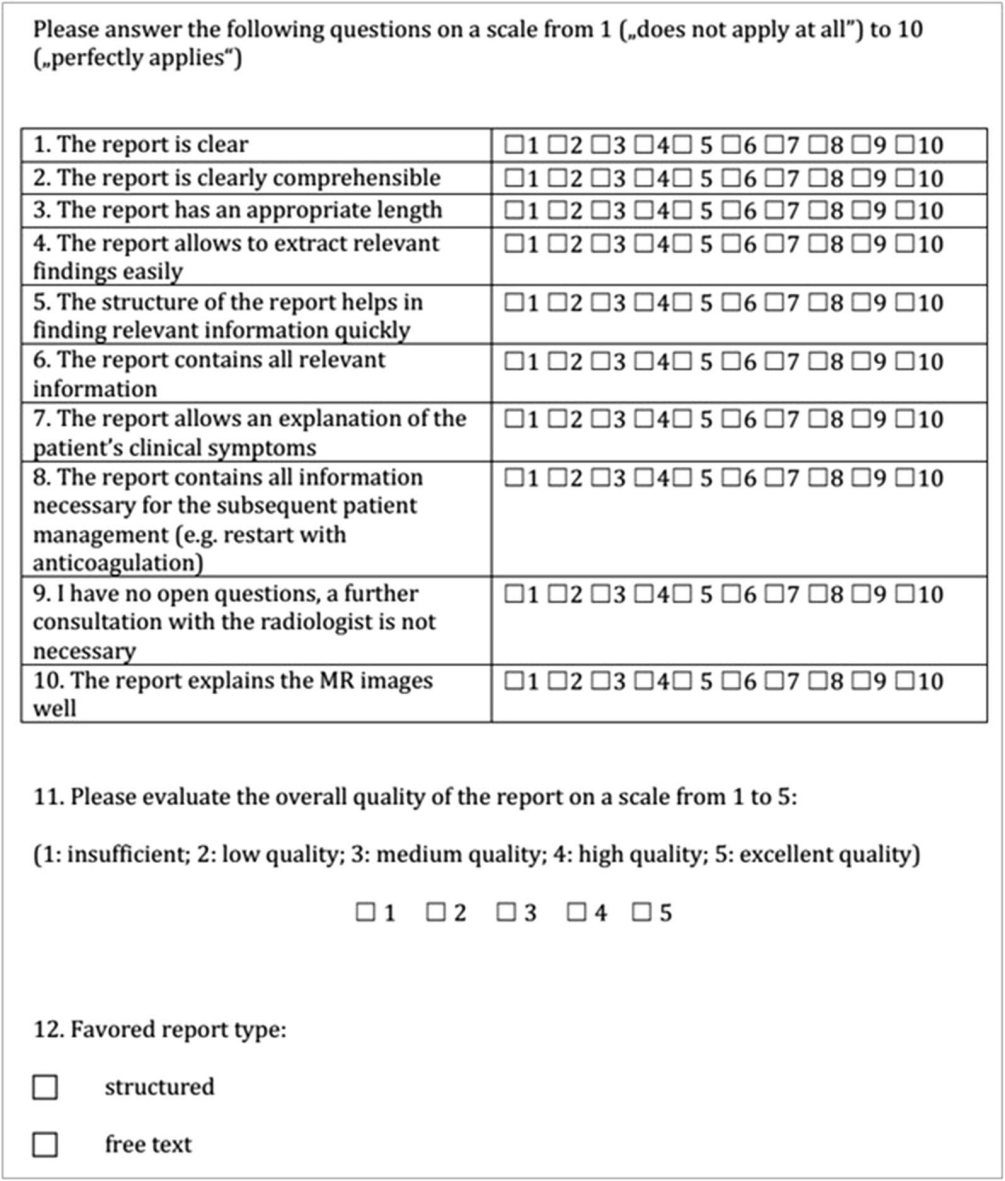
Questionnaire for neurologists

The reports and MR images were reviewed using a standard clinical PACS (Picture Archiving and Communication System) workstation (IDS7, Sectra AB, Linköping, Sweden). The images were then randomly ordered and pseudonymized, blinded for patient data including the original FTR, date of the examination. Two neuroradiologists, with six years (D.M.H.) and three years (S.M.) of experience in the interpretation of brain MRI examinations independently reviewed all images and established a SR for each patient using the described SR template. Afterwards, two neurologists, with seven (B.I.) and five (K.B.) years of experience evaluated and compared the original FTR and the newly generated SR. These two neurologists were not involved in the primary study design. The quality of these reports was then evaluated by the two neurologists on a Likert scale from 1 to 10 or from 1 to 5 depending on the question (Fig. 2). The statistical analysis for the differences between FTR and SR was performed using the Mann–Whitney U-test or the Chi-squared test. Statistical significance was assumed for p < 0.05.

### Statistical analysis

The statistical analysis was performed with the Statistical Package for Social Sciences v. 25 (SPSS, version 25, Chicago, IL, USA) and with Excel 2013 software (Microsoft, Redmond, WA, USA).

## Results

In this study, a total of 40 pseudonymized reports, 20 FTRs and 20 corresponding SRs, of 20 patients with AIS who had received an MRI of the brain three to five days after MT were included in the present study (Table 2).

**Table 2:** Baseline patient characteristics as well as procedure metrics and patient outcome

Gender [% female; n]	45 (9/20)
Age [years]	74.2 ± 11.1
Atrial fibrillation [%; n]	20 (4/20)
Hypertension [%; n]	25 (5/20)
Smoker [%; n]	85 (17/20)
Diabetes [%; n]	25 (5/20)
Hyperlipidemia[%; n]	40 (8/20)
Wake-up stroke	25 (5/20)
NIHSS on admission	12.8 ± 6.8
Vessel occlusion [%; n]	
Middle cerebral artery	70 (14/20)
Distal internal carotid artery	20 (4/20)
Basilar artery	10 (2/20)
Intravenous thrombolysis [%; n]	45 (9/20)
NIHSS after intervention [%; n]	6.6 ± 7.1
Time to recanalization [min]	207 ± 73
Successful recanalization [%; n]	85 (17/20)
Procedure related complications [%; n]	0 (0/20)
Intracranial hemorrhages [%; n]	0 (0/20)
Hemorrhagic transformation	
Hemorrhagic infarction type 1 [%; n]	20 (4/20)
Hemorrhagic infarction type 2 [%; n]	5 (1/20)
Parenchymal hemorrhage type 1 [%; n]	10 (2/20)
Parenchymal hemorrhage type 2 [%; n]	0 (0/20)

The questionnaire evaluation results of two neurologist experienced in treating AIS patients are depicted in Fig. 2. Both raters significantly favored the SR over the FTR regarding clarity (p < 0.001), comprehension (p < 0.001), apprehension of relevant findings quickly (p = 0.016), and the structure of the report in helping to find relevant information quickly (p = 0.005). Furthermore, the satisfaction with the content in regard to completeness of information necessary for immediate subsequent patient management (p < 0.001) and the explanation of the MR images were evaluated significantly higher for the SR by both raters (p = 0.020). Rater 1 stated that SR allow for a better explanation of the patient’s clinical symptoms (p = 0.003) and significantly reduce the number of open questions, which would have implied further consultation with the radiologist (p < 0.001). Furthermore, Rater 2 had the opinion that the SR contained all relevant information (p < 0.009). Rater 1 did not find the length of the two sorts of reports to be different, Rater 2 found the length of SR significantly more appropriate (p < 0.001).

The overall quality of the report was rated in favor of the SR in case of Rater 1 (p = 0.004) and, at least in tendency, in the case of Rater 2 (Table 3). The favored sort of report was the SR for both raters.

**Table 3:** Results of report quality ratings FTR versus SR

Questions	Rater 1		p value	Rater 2		p value
	FTR	SR		FTR	SR	
1. The report is clear	8.05 ± 0.51	9.05 ± 0.22	< 0.001*	6.80 ± 1.28	8.30 ± 0.66	< 0.001*
2. The report is clearly comprehensible	8.50 ± 0.61	9.10 ± 0.44	0.001*	7.35 ± 0.81	8.20 ± 0.83	0.001*
3. The report has an appropriate length	8.60 ± 0.50	8.40 ± 0.60	0.293	7.80 ± 1.36	5.75 ± 0.97	< 0.001*
4. The report allows to extract relevant findings easily	7.80 ± 0.60	8.95 ± 0.22	< 0.001*	7.05 ± 1.10	7.80 ± 0.83	0.016*
5. The structure of the report helps in finding relevant information quickly	7.60 ± 0.60	9.10 ± 0.31	< 0.001*	7.10 ± 0.97	8.05 ± 0.89	0.005*
6. The report contains all relevant information	8.60 ± 0.75	8.90 ± 0.56	0.113	6.80 ± 1.58	7.95 ± 1.05	0.009*
7. The report allows an explanation of the patient’s clinical symptoms	8.45 ± 0.69	9.00 ± 0.32	0.003*	7.30 ± 1.38	7.50 ± 0.51	0.881
8. The report contains all information necessary for subsequent patient management	7.90 ± 0.79	8.85 ± 0.37	< 0.001*	6.65 ± 1.42	8.60 ± 0.82	< 0.001*
9. I have no open questions, a further consultation with the radiologist is not necessary	8.55 ± 0.76	9.00 ± 0.32	< 0.001*	6.25 ± 1.45	6.75 ± 1.74	0.386
10. The report explains the MR images well	8.35 ± 0.74	9.05 ± 0.39	0.020*	7.10 ± 0.93	7.95 ± 0.75	0.003*
11. Please evaluate the overall quality of the report on a scale from 1 to 5	3.80 ± 0.41	4.0 ± 0.00	0.037*	3.60 ± 0.50	3.85 ± 0.49	0.127
12. Favored report type [%; n]	15 (3/20)	85 (17/20)	NA	45 (9/20)	55 (11/20)	NA

Results are depicted as mean values with standard deviation for Questions 1 to 11 and as percentage for question 12

Significance was assumed if p < 0.05 and highlighted by using italics

*FTR* free text report, *SR* structured report, *NA* not applicable

^*^p < 0.05

## Discussion

This study showed that SR substantially increase the report quality of brain MRI after MT in patients with AIS compared to FTR. To the best of our knowledge, no previous studies have analyzed this challenging issue. The data presented in this manuscript indicate that the use of SR leads to significantly improved report quality, completeness, and readability. These findings are consistent with previous studies, which have reported a superior report quality of SR in a number of diagnostic modalities and clinical settings [10, 14, 15]. One advantage of the SR developed in this study was that also small structures with high clinical impact for clinicians, as for example the internal capsule, are often not mentioned in FTR.

In ischemic stroke, the patterns of infarction, and possible complications, such as hemorrhage, infarct swelling or, brain tissue herniation, may be challenging to detect. The accurate diagnosis of the stroke patients post recanalization status is of high importance because it is essential for determining the correct treatment approach and risk stratification for further rehabilitation management. MRI is the imaging modality of choice for precise imaging of brain tissue and ischemia. Thus, physicians in charge of stroke care depend on high quality MRI reports. It has been shown for other diseases that SR can increase the report quality and, thereby, make the work of referring clinicians easier [15]. Besides a thorough clinical examination, accurate reporting is important for being able to provide the highest standards in diagnostics and therapy.

The evaluation results of the neurologist raters in this study showed that SR was significantly favored over the FTR regarding report clarity, comprehension, apprehension of relevant findings quickly, and structure. Furthermore, satisfaction with necessary information for the immediate subsequent patient management, explanation of the MR images as well as the overall favored sort of report was rated in favor of SR. These superior results of the SR over the FTR have been previously reported and discussed in a variety of other clinical fields [12, 14–16]. For example, SR of MRI in patients with primary rectal cancer achieved significantly higher satisfaction rates with report content and clarity as well as overall report quality in comparison to FTR. Potential benefits for surgical planning and interdisciplinary communication were deduced from this [15].

Furthermore, in our study the neurologists had less open questions resulting in a reduced need to consult the reporting radiologists. This may have a positive effect on the workflow on both sides.

The educational aspects of SR for radiology residents in training by, for example, containing all key features with correct wording have been elaborated before [17], which further underlines the importance of SR implementation in stroke care.

In this work, SRs were created by using a software tool that translates clickable decisions into predefined text phrases.

This study has several limitations. First, the retrospective design may have introduced some unexpected bias relative to the radiologist who interpreted the examinations prospectively. Only two clinicians were involved in the review of SR vs. FTR. However, we tried to minimize this confinement by the strict independence of our reviewers. As the reviewers were not involved in the study design at the beginning, this may minimize a bias towards one or the other type of reporting. Furthermore, we did not measure the time needed for producing the SR and FTR was not included, simply because the study was retrospective and times for FTR generation were not available. Further studies should investigate the impact of SR in stroke MRI on clinical practice and workflows prospectively.

## Conclusions

This study suggests that structured reports compared to free-text reports of brain MRI examinations in acute ischemic stroke patients which have been treated using mechanical thrombectomy is favored by stroke neurologists regarding clarity, comprehensibility, quick apprehension of relevant findings and the structure of the report. Structured reports potentially facilitate the stroke teams planning regarding next treatment steps and may lead to a higher satisfaction level of referring neurologists.

## Data Availability

The datasets used and/or analysed during the current study are available from the corresponding author on reasonable request.

## Abbreviations

AIS: Acute ischemic stroke
Art. TOF: Arterial time-of-flight MR angiography
AT: Acquisition time
CT: Computer tomography
CTA: CT angiography
DWI: Diffusion weighted imaging
FLAIR: Fluid attenuation inversion recovery
FTR: Free-text report
MR: Magnetic resonance
MRI: Magnetic resonance imaging
MT: Mechanical thrombectomy
NA: Not applicable
NIHSS: National Institutes of Health Stroke Scale
SR: Structured report
T: Tesla
TE: Time to echo
TR: Time to repeat
TI: Inversion time
